# Roles of Argonautes and Dicers on *Sclerotinia sclerotiorum* Antiviral RNA Silencing

**DOI:** 10.3389/fpls.2019.00976

**Published:** 2019-07-30

**Authors:** Achal Neupane, Chenchen Feng, Pauline K. Mochama, Huma Saleem, Shin-Yi Lee Marzano

**Affiliations:** ^1^Department of Biology and Microbiology, South Dakota State University, Brookings, SD, United States; ^2^Department of Agronomy, Horticulture and Plant Science, South Dakota State University, Brookings, SD, United States

**Keywords:** RNA pesticide, argonautes, dicers, mycovirus, *Sclerotinia sclerotiorum*, RNA silencing, tRNA halves

## Abstract

RNA silencing or RNA interference (RNAi) is an essential mechanism in animals, fungi, and plants that functions in gene regulation and defense against foreign nucleic acids. In fungi, RNA silencing has been shown to function primarily in defense against invasive nucleic acids. We previously determined that mycoviruses are triggers and targets of RNA silencing in *Sclerotinia sclerotiorum*. However, recent progresses in RNAi or dsRNA-based pest control requires more detailed characterization of the RNA silencing pathways in *S. sclerotiorum* to investigate the utility of dsRNA-based strategy for white mold control. This study elucidates the roles of argonaute enzymes, *agl*-2 and *agl*-4, in small RNA metabolism in *S*. *sclerotiorum*. Gene disruption mutants of *agl*-2 and *agl*-4 were compared for changes in phenotype, virulence, viral susceptibility, and small RNA profiles. The Δ*agl*-2 mutant but not the Δ*agl*-4 mutant had significantly slower growth and virulence prior to virus infection. Similarly, the Δ*agl*-2 mutant but not the Δ*agl*-4 mutant, showed greater debilitation under virus infection compared to uninfected strains. The responses were confirmed in complementation studies and revealed the antiviral role of *agl*-2. Gene disruption mutants of *agl*-2, *agl*-4, Dicer-like (*dcl*)-1, and *dcl*-2 did not change the stability of the most abundant endogenous small RNAs, which suggests the existence of alternative enzymes/pathways for small RNA biogenesis in *S. sclerotiorum.* Furthermore, *in vitro* synthesized dsRNA targeting *agl*-2 showed a significantly reduced average lesion diameter (*P* < 0.05) on canola leaves with *agl*-2 down-regulated compared to controls. This is the first report describing the effectiveness of RNA pesticides targeting *S*. *sclerotiorum* RNA silencing pathway for the control of the economically important pathogen.

## Introduction

RNA silencing is a transcriptional and post-transcriptional suppression of gene expression. One of the roles that RNA silencing plays has long been identified as an adaptive defense mechanism against foreign nucleic acids, including viruses in animals, fungi, and plants (Waterhouse et al., 2001; Baulcombe, 2004, 2005). Unlike in animals and plants, the evolved RNA silencing in fungi to date has demonstrated that it is almost dispensable for endogenous gene regulation because gene disruption mutants often grow just fine. Instead, only when the mutants of RNA silencing genes are under virus infection, the antiviral role of those genes play then become evident (Segers et al., 2007; Zhang et al., 2014; Yu et al., 2018). However, studies of *Neurospora crassa* and other filamentous fungi have revealed diverse small RNA biogenesis pathways, suggesting that fungi adapt RNAi silencing pathways for several cellular processes with some of the RNA silencing genes playing dual roles (reviewed in Dang et al., 2011). On the other hand, fungal RNA silencing genes can also have redundant functions, such as antiviral, processing of dsRNA or transgenes (Catalanotto et al., 2004; Wang et al., 2016; Yu et al., 2018).

*Sclerotinia sclerotiorum* is a devastating plant fungal pathogen that causes up to 100% yield losses in crop production affecting a wide array of crops (Heffer Link and Johnson, 2007). Recent studies demonstrated that cross-kingdom RNA silencing can be blocked to control *Botrytis cinerea* which is closely related to *S*. *sclerotiorum* (Amselem et al., 2011). The virulence of *B*. *cinerea* can be greatly suppressed by silencing both *B*. *cinerea* Dicers at the same time (Wang et al., 2016). A similar observation was made in *S. sclerotiorum* following simultaneous disruption of both its Dicers to result in reduced pathogenicity (Mochama et al., 2018). Therefore, RNA silencing pathway has great potential to be manipulated to control fungal pathogens. As *S. sclerotiorum* has two predicted Argonautes (GenBank accession numbers Ss1G_00334 and Ss1G_11723), it is intriguing whether corresponding argonaute genes affect *S*. *sclerotiorum* virulence and whether it could add to the tool box of disease control with other novel strategies.

The Argonaute protein family constitutes four domains, N-terminal domain, Mid domain, and RNA-binding domains known as PAZ domains, and slicer domains known as PIWI domains (Poulsen et al., 2013). Argonaute proteins stabilize small dsRNA molecules produced by Dicer proteins to form RNA-induced silencing complexes (RISC) which are involved in post-transcriptional gene silencing or RNA-induced transcriptional silencing complexes involved in transcriptional gene silencing including chromatin modification in animals, plants, and insects (Irvine et al., 2006). When small dsRNA molecules produced by Dicers are incorporated into these effector complexes, one strand of the RNA molecule is removed and the remaining strand guides the complex to complementary RNA sequences which are subsequently cleaved by the Argonaute RNase H-like activity (Qihong et al., 2009).

Argonaute homologs have been identified in various fungi and they differ in function and number. The basal fungus, *Mucor circinelloides*, has three argonaute genes while *Cryphonectria parasitica* has four argonaute genes and *Colletotrichum higginsianum* has two (Qihong et al., 2009; Trieu et al., 2015; Campo et al., 2016). QDE-2 is a fungal argonaute homolog in *N*. *crassa* involved in quelling-the silencing of repetitive sequences such as transgenes (Fulci and Macino, 2007). In *N*. *crassa*, a separate silencing pathway called meiotic silencing of unpaired DNA (MSUD) has been characterized, and *N*. *crassa* RNA silencing components not involved in quelling have been shown to be involved in this pathway (Fulci and Macino, 2007). Similarly, in other fungi, not all components of the RNA silencing machinery are involved in RNA silencing mediated viral defense mechanisms. In *Fusarium graminearum*, only one of two argonaute genes, FgAgo1, is important in RNA silencing of viral nucleic acids (Yu et al., 2018) while in *C*. *parasitica* only *agl*-2 is required for antiviral RNA silencing, and in *C*. *higginsianum*, *agl*-1 but not *agl*-2 is essential for antiviral RNA silencing (Qihong et al., 2009; Trieu et al., 2015; Campo et al., 2016). The primary functions of the other gene homologs have not been fully characterized. As *S*. *sclerotiorum* are predicted to have two argonaute genes, *agl*-2 and *agl*-4, it presents a potential strategy to impede the proper small RNA processing after characterizing the roles of argonautes in *S*. *sclerotiorum*.

The goals of this study were to determine the function of argonaute genes in endogenous small RNA processing and defending virus infection in *S*. *sclerotiorum*, and as a proof of concept, to demonstrate a control strategy from silencing a specific argonaute gene. To achieve them, we made gene displacement mutants of argonaute genes in this study and transfected *S*. *sclerotiorum* with a RNA virus (SsHV2-L) and compared the changes in morphology and pathogenicity. Gene displacement mutants revealed that only *agl*-2 is important in vegetative growth, as well as antiviral defense, whereas the biological function of *agl-4* remains unknown. We further established the application of dsRNA externally targeting *agl*-2 as an RNA pesticide to slow the infection in a dose-dependent manner.

## Materials and Methods

### Fungal Culture Strains and Conditions

The wild type strain, DK3, of *S*. *sclerotiorum* was grown on potato dextrose agar (PDA) (Sigma) at 20–22Δ. The gene displaced mutants of *Δagl*-2 and *Δagl*-4 strains were grown on PDA amended with hygromycin B (Alfa Aesar) at 100 μg/mL as selection. Dicer mutants were produced in our previous study (Mochama et al., 2018).

### Gene Disruption of *agl*-2 and *agl*-4

**FIGURE 1:**
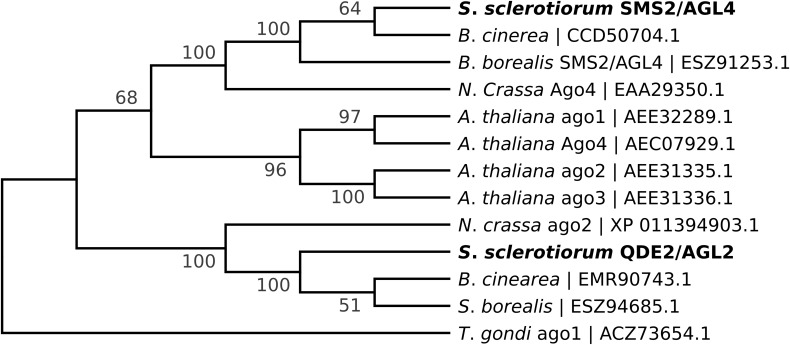
Phylogenetic analysis (Maximum Likelihood tree) of *agl*-2 and *agl*-4 genes depicting the relationships between the protein sequences from *S*. *sclerotiorum* (in bold), along with their orthologs in *B*. *cinerea*, *S*. *borealis*, *N*. *crassa* and *Arabidopsis thaliana*. The *Toxoplasma gondii agl*-1 amino acid sequence was used as an outgroup. Bootstrap consensus was calculated based on 100 bootstrap replicates using Mega 7.0 (Kumar et al., 2016).

*Sclerotinia sclerotiorum* argonaute-like genes were predicted based on homology to those identified in *N. crassa* (Laurie et al., 2012). Published sequences of Ss1G_00334 and Ss1G_11723 in the GenBank (NCBI) are the putative argonaute genes coding for QDE-2/AGO-2 and SMS-2/AGO-4 in *N*. *crassa*, respectively (Figure 1). Argonaute genes were displaced by the hygromycin phosphotransferase gene (*hph*) using the split-marker homologous recombination cassettes as described before (Mochama et al., 2018). To generate the Δ*agl*-2 gene displacement mutant, a 1.5 kb 5′ flanking arm of *agl*-2 was PCR-amplified by primers F1-AGO2 and F2-AGO2 and a 1.5 kb 3′ flanking arm of *agl*-2 was PCR-amplified by primers F3-AGO2 and F4-AGO2. The *hph* gene was amplified from pCSN43 (Fungal Genetics Stock Center) using primers PtrpC – HY and YG – TrpC to give two amplicons (1.2 and 1.3 kb) each containing part of the *hph* marker gene with an overlap (Table 1). The 5′- flank of the *Δagl*-2 gene was then connected to the partial *hph* amplicon containing P*trp*C and the 3’ flank was connected to the *hph* amplicon containing T*trp*C using the overlap extension PCR method. Eventually, an *agl*-2 gene deletion construct that included 1 kb of identical sequence to the 5′ flanking arm of the gene and 812 bp of the 3′ flanking arm sequence was derived. A similar procedure was used to generate the *agl*-4 deletion construct with 805 bp of sequence identical to the 5′ flanking arm and 1.1 kb of the 3′ flanking arm of *agl*-4.

**TABLE 1:** Primer used in this study.

**Primer Name**	**Sequence**	**Note**
F1-AGO2	TGGTGAATTGTGAGTT GAATGGTG	*agl*-2 KO
F2-AGO2	ACCCAATTCGCCCTATAGT GAGTCGTGCTGCTGGA TCAAAAGACAT	*agl*-2 KO
F3-AGO2	AAGCCTACAGGACACACA TTCATCGTAGGTACCT GGTCATACCTTCCGCAT	*agl*-2 KO
F4-AGO2	CAGGTCCAAGTCCT GTCCAC	*agl*-2 KO
YG-F	CGTTGCAAGACCTGC CTGAA	Both KO
PtrpC-F	ACGACTCACTATAGGG CGAATTGGGT	Both KO
TtrpC-R	TACCTACGATGAATGTGTG TCCTGTAGGCTT	Both KO
HY-R	GGATGCCTCCGCTCGA AGTA	Both KO
F1-AGO2-nested	GTTTGCAACAATC GCAGGTG	*agl*-2 KO
F4-AGO2-nested	TCTCCAACCAG CTACCGATG	*agl*-2 KO
F1-AGO4	TTTGGTCCAGG CCTTGGTTT	*agl*-4 KO
F1-AGO4-nested	TTTTCACAACGG GTTTGGGC	*agl*-4 KO
F2-AGO4	ACCCAATTCGCCCTATAGT GAGTCGTGAGCCATTAGCTTGG ATATTCGCA	*agl*-4 KO
F3-AGO4	AAGCCTACAGGACACA CATTCATCGTAGGTAAGTGCCT TCATATCATAATCCTCC	*agl*-4 KO
F4-AGO4	AAGGTTCGTCGGTT GGTAGT	*agl*-4 KO
F4-AGO4-nested	CCCTACTTGTCCC ACGTGAT	*agl*-4 KO
F1-COMP-AGO2	ATAATAGCGGCCGCCGGA TAAACTGCCTCTTCGC	*agl*-2 Complementation
F4-COMP-AGO2	TTTTACTGGATCCTCCT GTCCACAATCCCACAA	*agl*-2 Complementation
Ss-Ago2-T7p-1898F	TAATACGACTCACTATAG GGAGATGAATCTACA AGCCGTGCTG	dsRNA synthesis targeting *agl*-2
Ss-Ago2-T7p-2065R	TAATACGACTCACTATAGG GAGATGAGAGGTA GCCGCTTCATT	dsRNA synthesis targeting *agl*-2
Ss-Ago4-T7p-F	TAATACGACTCACTATAGGGAGA TTTGGTTGCAAAATCGATCA	dsRNA synthesis targeting *agl*-4
Ss-Ago4-T7p-R	TAATACGACTCACTATAGGGA GATTGCTTGCTTTGTTGACCAG	dsRNA synthesis targeting *agl*-4

### Fungal Transformation

PEG-mediated transformation method was used to transfer the gene deletion cassettes into *S. Sclerotiorum* DK3 protoplasts as described before (Mochama et al., 2018). Fungal DNA was extracted from mycelia and PCR-amplified by the use of primers- F1, F4, F1, HYR, YG2, and F4 to ascertain that argonaute genes were each displaced by the *hph* gene, confirmed by Sanger sequencing. Because *S*. *sclerotiorum* does not produce conidia, repeated hyphal tipping and nested PCR were necessary to derive a monokaryotic line of each targeted gene disruption to avoid mixed results from heterokaryotic mutants.

### Complementation

To complement *agl*-2, protoplasts from the Δ*agl*-2 strain was transformed with a bialaphos plasmid (pBARKS-1) cloned to express the full *agl*-2 gene flanked by 2.8 kb of 5′-upstream and 1.5 kb of 3′-downstream sequences to include the corresponding promoter and terminator. The *agl*-2 gene and flanking arms were amplified from the DNA extract of DK3 using primers F1-COMP-AGO2 and F4-COMP-AGO2 (Table 1) and inserted into the *Not*I and *BamHI* sites of pBARK-1 downstream to PTrpC and bialaphos resistance gene, *bla*1. Protoplasts and PEG-mediated transformation were the same as described earlier, except that the regeneration media was now supplemented with bialaphos at 10 μg/mL for selection. Multiple transformants were selected and hyphal-tipped several times to fresh PDA plates amended with bialaphos. PCR amplification using *agl*-2 specific primers confirmed the ectopic integration of the gene, and then four transformants were compared for the morphology on PDA between the virus-free and SsHV2-L virus-infected complemented strains.

### Phenotypic Characterization of Gene Deletion Mutants

At least five replications each of DK3, Δ*agl*-2, and Δ*agl*-4 cultures with or without SsHV2 infections were compared for the phenotypes. Hyphal diameter was measured daily as described before (Mochama et al., 2018). A 5-mm plug was placed on a freshly cut canola leaf. More than three replicates of the leaves were inoculated on moist paper towels in covered petri dishes kept on a lab bench at room temperature. Hyphal area was measured daily at 24, 48, and 72 h post-inoculation.

### *In vitro* dsRNA Synthesis, Inoculation, and Confirmation of Silencing by RT-qPCR

PCR amplification of the *agl*-2 target was performed using gene-specific primers with T7 promoter sequence added on both the forward primer and the reverse primer. Primers Ss-Ago2-T7p-1898F and Ss-Ago2-T7p-2065R were used (Table 1). dsRNAs were synthesized using MEGAscript T7 Transcription kit (Invitrogen) following the manufacturer’s procedure. To compare the suppressing effect of dsRNA on fungal pathogenicity, a 2 days actively growing plug in 3-mm diameter taken from the margin of a colony of *S*. *sclerotiorum* DK3 was placed on each canola leaf. Six replications each of different doses of abovementioned dsRNA at 200, 400, and 800 ng was pipetted to surround an agar plug in the volume of 20 μl, taking reference from the dosage of 800 ng/20 μl published by Wang et al. (2016). As controls, the same volume of water, as well as dsRNA targeting *agl*-4 were pipetted to surround the agar plug on canola leaves. dsRNA targeting *agl*-4 was produced the same way as that targeting *agl*-2 but with primers Ss-Ago4-T7p-F and Ss-Ago4-T7p-R. The lesion was measured length wise and at right angle across again to obtain an average for a representative diameter 2 days post-inoculation. The data was statistically analyzed using paired *t*-test; and using one-way ANOVA (for three or more samples), and when significant effect was determined, Tukey’s HSD test was performed to compare all pairs of means.

Only the lesions from 200 and 400 ng/20 μl were cut out to extract for total RNA using RNeasy Plant Mini Kit (Qiagen) since 800 ng/20 μl treatment does not produce lesions. RT-qPCR was performed to confirm the silencing of *agl*-2 gene in a dose-response manner using Luna Universal One-Step RT-qPCR Kit (NEB) following the manufacturer’s protocol. The comparative CT method (ΔΔCT method) was used to analyze the data. The expression levels of *agl*-2 and an endogenous control (actin) were evaluated with three biological replicates and four technical replicates each. The statistical significance of observed fold-difference was analyzed by ANOVA and Tukey’s test for pair-wise means separation.

### Small RNA Libraries Preparation and Analysis of the Sequencing Results

MirVana miRNA Isolation kit (Thermo Fisher Scientific) was used to extract small RNAs from 4-day-old mycelia. NEBNext small RNA Library Kit (NEB, Ipswich, MA, United States) was used to construct the libraries for sequencing. The libraries were barcoded, pooled in a single lane for 50-nt single-end reads sequencing on an HiSeq4000 at the Roy J. Carver Biotechnology Center, UIUC. Three replicates of samples from SsHV2-L virus-infected DK3 as well as four replicates (two virus-infected and two virus-free mutants) each of *Δdcl*-1, *Δdcl*-2, *Δagl*-2, and *Δagl*-4 samples were sequenced. Adaptors were trimmed by BBMap tools (Bushnell, 2014). ShortStack (Axtell, 2013) was used to identify loci producing sRNAs by clustering. The number of reads aligned to *S*. *sclerotiorum* and SsHV2-L genomes were computed using bowtie (Langmead et al., 2009), and further downstream analysis were performed using in-house Perl and R scripts. tRNA encoding genes were predicted by tRNAscan-SE (Lowe and Chan, 2016).

## Results

### Disruption Mutants of Argonaute-Like Genes Were Generated

Argonaute-like genes were disrupted directly from wild-type strain DK3 using the same approach as described before (Mochama et al., 2018). Disruption was screened by PCR amplification using F1 and F4 primers and DNA extracts from multiple transformants as the templates to rule out ectopic integration of the *hph* gene. Sanger sequencing of the PCR amplicons confirmed the integration. Once a monokaryotic mutation was obtained by hyphal-tipping and confirmed by PCR that the target genes were completely deleted, further characterizations of the mutants were carried out.

### Effect of Argonaute-Like Genes Disruption on Phenotype

**FIGURE 2:**
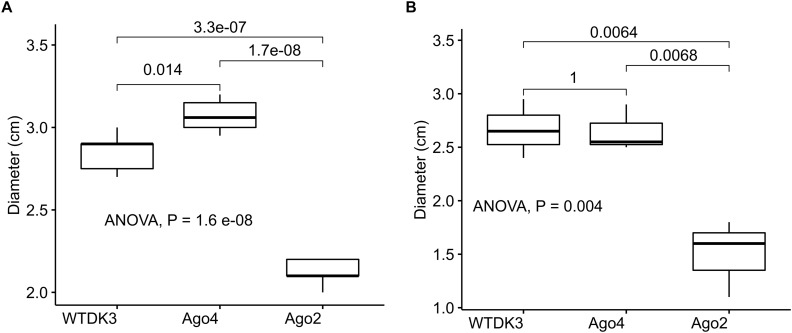
Average hyphal diameter of *S. sclerotiorum* wild type and argonaute gene disruption mutants (*Δagl*-2 and *Δagl*-4) grown **(A)** on PDA for 1 dpi, and **(B)** on detached canola leaves 2 dpi.

**FIGURE 3:**
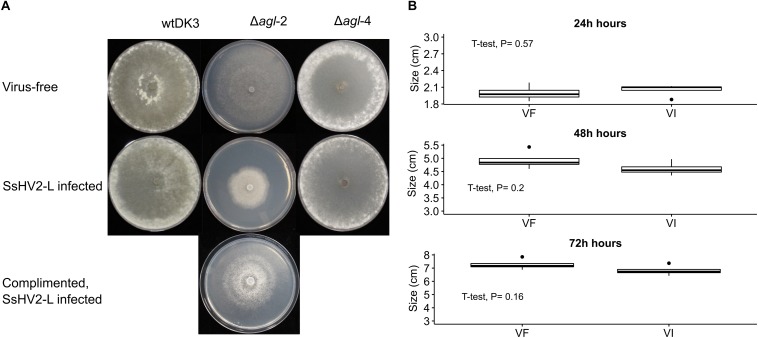
Colony morphology of virus-free and virus-infected gene deletion mutants. (Top row) Virus-free DK3, Δ*agl*-2 and Δ*agl*-4. (Bottom row) wild-type and mutant strains infected with hypovirus SsHV2-L. Cultures were grown for **(A)** 4 days on PDA. The virus-infected *agl*-2 mutant displays significantly slower growth and altered colony morphology. **(B)** Comparison of the complemented strains with and without virus infection (*P* > 0.05).

**FIGURE 4:**
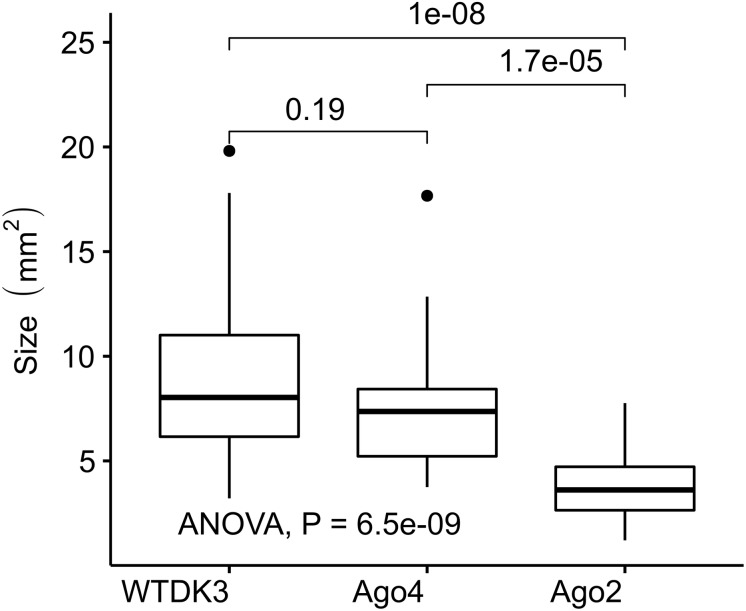
Comparison of sclerotial morphology in wild-type DK3 and mutant strains, *Δagl*-2 and *Δagl*-4. The *Δagl*-2 mutant produces smaller sclerotia on average.

The colony morphology including the growth rate and the size of the sclerotia of the argonaute mutants and the wild-type strain DK3 on PDA were compared. Single mutant Δ*agl*-4 and DK3 exhibited similar growth rates, whereas the Δ*agl*-2 gene displacement mutant exhibited significantly slower growth at 24 h measured by the diameters of hyphal growth (*P* < 0.05) (Figure 2A). As shown in Figure 3, at 4 days post-inoculation (dpi) (Figure 3A), no change in growth rate was observed in Δ*agl*-4, whereas Δ*agl*-2 mutant shows a slower growth and a reduction in the size of sclerotia produced (Figure 4). After multiple times of hyphal-tipping, four *agl*-2 complemented transformants were assayed. The complemented strains exhibited a reversal of phenotype in antiviral defense (Figure 3B).

### Effects of Argonaute-Like Gene Disruptions on *S*. *sclerotiorum* Pathogenicity

**FIGURE 5:**
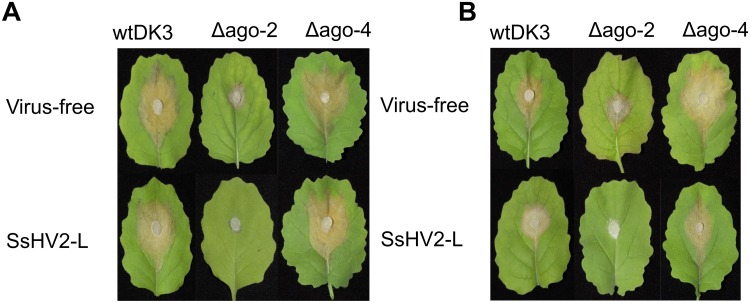
Virulence assays on detached canola. Plugs were taken from the edge of actively growing DK3, *Δagl*-2, and *Δagl*-4 cultures and inoculated onto detached leaves. Lesion size was measured **(A)** 2 dpi, **(B)** 3 dpi.

The virulence of *S. sclerotiorum* argonaute mutants was evaluated by inoculating detached leaves with agar plugs of mycelia. Lesion size data was collected at 1, 2, and 3 dpi showed that no difference in the lesion size produced by the mutant Δ*agl-*4, but a significantly smaller lesion produced by Δ*agl*-2 mutant compared to those produced by DK3 (Figures 2B, 5) (*P* < 0.05).

### Effects of Argonaute Gene on Antiviral Defense

To examine the effect of viral infection on strains containing null-mutations of *agl*-2 and *agl*-4, mutants were transfected through hyphal fusion with SsHV2-L infected mycelia. As shown in Figure 3, no significant differences were observed in growth and morphology in the *agl*-4 mutant infected with the mycovirus compared to virus-infected DK3, whereas the *agl*-2 mutant showed a significantly debilitated growth (Figures 3, 5) (*P* < 0.05).

### *In vitro* Synthesized dsRNA Targeting *agl*-2 Shows Reduced Virulence of *S*. *sclerotiorum*

**FIGURE 6:**
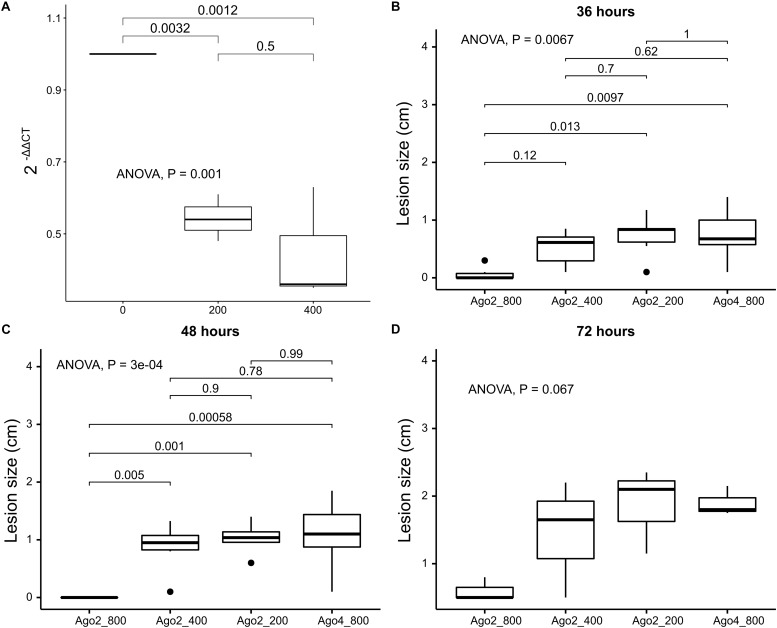
Effects of external RNA pesticide on inhibiting *S. sclerotiorum* from causing lesions on canola leaves comparing dsRNA targeting *agl*-2 at 800 ng/20 μl, 400 ng/20 μl, 200 ng/20 μl, dsRNA targeting *agl*-4 at 800 ng/20 μl as control (from left to right), confirmed by **(A)** RT-qPCR with reduced expression levels of *agl*-2 at 200 and 400 ng, and lesion comparison at **(B)** 36, **(C)** 48, and **(D)** 72 h post-inoculation.

Once we determined that *agl*-2 plays an important role in endogenous small RNA processing, exemplified by a debilitated growth even without virus infection, the *agl*-2 was then targeted using *in vitro* synthesized dsRNA constructs in order to disrupt the fungal small RNA processing. RT-qPCR confirmed that *agl*-2 was silenced at the level of 800 ng in 20 μl volume but not at the lower doses of 200 or 400 ng (Figure 6A). As shown in Figures 6B–D, strains in which *in vitro* 800 ng of dsRNA was applied externally to target *agl*-2 exhibited a slower spread on canola leaves up to 3 days post-infection compared to lower doses at 200 and 400 ng applied or the targeting of *agl*-4 by the corresponding dsRNA (Supplementary Material).

### Profiles of sRNAs in Distinct *S*. *sclerotiorum* Strains

The raw sequence reads were uploaded to NCBI SRA database under accessions SRR8844548 (WT-1), SRR8844549 (WT-2), SRR8785208 (WT-3), SRR8785205 (*Δ*dcl1-1), SRR8785204 (*Δ*dcl1-2), SAMN12129781 (*Δ*dcl1-VF1), SAMN12129782 (*Δ*dcl1-VF2), SRR8785203 (*Δ*dcl2-1), SRR8785202 (*Δ*dcl2-2), SAMN12129783 (*Δ*dcl2-VF1), SAMN12129784 (*Δ*dcl2-VF2), SRR8785201 (*Δ*agl2-1), SRR8785200 (*Δ*agl2-2), SAMN12129777 (*Δ*agl2-VF1), SAMN12129778 (*Δ*agl2-VF2), SRR8785199 (*Δ*agl4-1), SRR8785198 (*Δ*agl4-2), SAMN12129779 (*Δ*agl4-VF1), and SAMN12129780 (*Δ*agl4-VF2). Table 2 summarized the numbers of aligned small RNA sequence reads from the mutants and the WT sample that passed the ShortStack filtering parameters to allow the clustered reads for downstream analysis.

**TABLE 2:** Numbers of aligning small RNA sequence reads from hypovirus-transfected wild type and mutants of *Sclerotinia sclerotiorum*.

	**Read counts**	**Percent of aligned reads**					
		
**Samples**	**Filtered read**	**IGR (% of tRNA derived small RNA)**	**CDS**	**Retrotransposons**	**Mitochondria**	**rRNA**	**Other**
Ago2_SsHV2L_1	6510462	46.4 (42.7%)	10.6	3.3	5.7	5.6	28.5
Ago2_SsHV2L_2	6599083	46.7 (46.73%)	9.3	2.9	5.6	5.8	29.7
Ago2_VF_1	5895665	48.4 (31.46%)	12.7	4.4	5.6	4.3	24.6
Ago2_VF_2	5646987	49.1 (32.7%)	13.5	4.8	5.2	3.9	23.5
Ago4_SsHV2L_1	15140898	58.1 (68.9%)	5.9	2.2	2.8	1.9	29.1
Ago4_SsHV2L_2	8083962	49.4 (56.8%)	8.2	3.0	4.5	8.0	26.9
Ago4_VF_1	18251848	80.1 (78.6%)	3.9	0.5	1.6	4.2	9.7
Ago4_VF_2	13039613	83.0 (80.6%)	3.5	0.5	1.8	2.8	8.4
Dcl1_SsHV2L_1	6932872	32.9 (77.6%)	2.2	0.2	1.7	24.9	38.1
Dcl1_SsHV2L_2	7040569	52.5 (62.7%)	5.3	2.3	2.6	9.2	28.1
Dcl1_VF1	9213301	50.4 (57.8%)	6.3	1.5	4.5	8.0	29.4
Dcl1_VF2	9087471	32.7 (28.6%)	5.6	0.9	4.2	19.8	36.8
Dcl2_SsHV2L_1	14427517	43.6 (62.5%)	5.6	2.3	4.6	4.9	38.9
Dcl2_SsHV2L_2	11097593	43.2 (59.2%)	6.7	3.0	4.2	5.5	37.3
Dcl2_VF1	17041328	40.6 (35.4%)	9.8	3.4	4.7	6.7	34.7
Dcl2_VF2	13619703	50.0 (59.2%)	5.6	1.8	4.7	7.2	30.8
WTDK3_SsHV2L_1	15565013	63.7 (76.0%)	5.1	1.7	3.1	3.4	23
WTDK3_SsHV2L_2	14313982	39.2 (41.4%)	9.4	4.4	2.8	1.2	43
WTDK3_SsHV2L_3	14553278	40.8 (25.4%)	11.6	4.5	8.5	2.8	31.8

**FIGURE 7:**
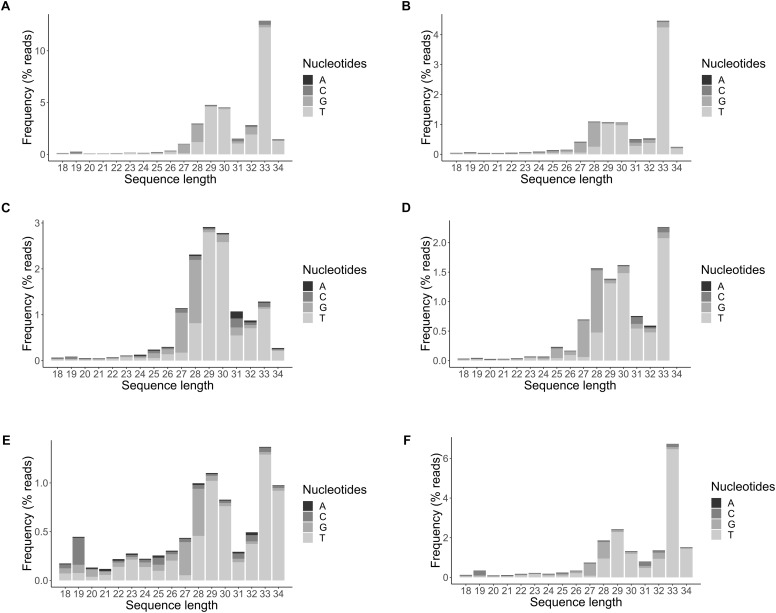
tRNA-derived small RNAs profiled for their size distribution in panel **(A)** wild type strain DK3, **(B)** Δ*dcl*-1, **(C)** Δ*dcl*-2, **(D)** Δ*dcl*-1/*dcl*-2, **(E)** Δ*agl*-2, **(F)** Δ*agl*-4.

A major portion of endogenous small RNAs were found to represent the same small RNAs identified from our previous study (Lee Marzano et al., 2018), predominantly tRNA halves (tRFs): tRF5-Glu(GAA), 5′-TCCGAATTAGTGTAGGGGTTAACATAACTC-3′, and tRF5-Asp(GAC), 5′-TCTTTGATGGTCTAACGGTCATGATTTCC-3′, derived from tRNAs delivering glutamic acid and aspartic acid at 1.1 and 1.3% of total filtered reads in average, respectively. Homologs of the abovementioned two tRFs range in size from 29 to 35 bases with a major peak at 33 nt as TCTTTGATGGTCTAACGGTCATGATTTCCGTCC (Figures 7A–D) (underlined are the bases when expanding the size of small RNAs up to 34 nt long). tRF5-Glu(GAA) is predicted to be produced from SS1G_14562 and SS1G_14600 on chromosomes 7 and 4, respectively, whereas tRF5-Asp(GAC) is produced from SS1G_14527 on chromosome 14. The clustering result showed that these two small RNAs were mapped solely to intergenic regions. The BLASTn search (Altschul et al., 1990) indicates their homology to specific loci on chromosomes 4, 7, 14, and 16 for tRF5-Glu(GAA), and on chromosomes 1, 5, 11, 12, and 14 for tRF5-Asp(GAC). These small RNAs and their homologs are derived from mature tRNAs and contribute to the major peak at 29 or 33 nt. Compared to the wild type (Figure 7A), the productions of the two species were not affected by either single dicer mutations (Figures 7B,C), nor double dicer mutations (Figure 7D) (Mochama et al., 2018), and the stability of these small RNAs were not drastically affected by argonaute mutations (Figures 7E,F). Therefore, the reanalyzed data of double dicer mutant (previously published in Mochama et al., 2018) revealed that the production of these tRFs were not produced by either dicer.

## Discussion

Studies conducted on a number of fungal species have uncovered robust RNA silencing mechanisms with important roles in fungal antiviral defense. Similarly, this study elucidates the RNA silencing mechanisms in *S*. *sclerotiorum* and establishes the significant roles played by Argonaute-like genes in this devastating plant pathogenic fungus. Primarily, these findings clearly demonstrate that while the wild-type strain displayed reduced virulence following SsHV2-L virus infection (Marzano et al., 2015), RNA-silencing-deficient mutants [specifically *Δagl*-2 mutant in this study, and previously reported *Δdcl*-1*/dcl*-2 double mutant (Mochama et al., 2018)] displayed an even more significantly debilitated growth and reduced virulence under virus infection.

The slower growth of *Δagl*-2 without virus infection also suggested that *agl*-2 contributes to cellular gene regulation through the prevention of RISC formation with endogenous small RNAs. Specifically, we found that the deletion of *agl*-2 gene but not *agl*-4 resulted in compromised growth and virulence prior to virus infection, suggesting the contributions made by *agl*-2 to physiological and developmental processes. The *agl*-2 mutant exhibited slower growth, smaller sclerotia, and reduced virulence. Therefore, the changes observed in the *agl*-2 mutant may be attributed to a significant reduction in small RNA loading and stabilization of endogenous small RNAs. As expected, size distribution of small RNAs is not greatly affected upon the deletions of *agl*-2 or *agl*-4 genes when the Dicers are functional.

The great debilitation observed in the *Δagl*-2 mutant caused by virus infection was not detected in the virus-infected *Δagl*-4. This suggests that the AGL-2 protein is solely responsible for incorporating vsiRNAs into the RISC complex as part of the viral RNA silencing mechanism leading to the silencing of viral RNA. Argonaute proteins have been shown to associate with vsiRNAs in plants to target complementary viral mRNAs and in some cases host genes as well (Mengji et al., 2014; Carbonell and Carrington, 2015). miRNA-like molecules with possible gene regulation functions have been found to associate with fungal Argonaute proteins like the QDE-2 protein in *N. crassa* (Lee et al., 2010). Our study suggests that AGL-2 protein in *S*. *sclerotiorum* may also contribute to endogenous gene regulation. While AGL-4 protein’s function remains unknown, it is likely to play important roles including miRNA degradation (Sheu-Gruttadauria et al., 2019). Moreover, as single argonaute mutants do not have drastic changes in small RNA stability, this suggests possible functional redundancy in the two Argonautes.

Single gene disruption mutants of *dcl*-1, *dcl*-2, *agl*-2, *agl*-4, and double dicer mutants of *dcl*-1/*dcl*-2 did not alter the accumulation of tRFs, which suggests the existence of alternative enzymes or pathways for the biogenesis of this class of small RNA in *S*. *sclerotiorum*. Other endonuclease exist in *S*. *sclerotiorum*, such as RNaseL-like endonuclease that share similarities with yeast Ire1p proteins which are said to be involved in fungal mRNA splicing (Dong et al., 2001). Another endonuclease, Rny1 in yeast, is a ribonuclease T2-like precursor, and disruption of Rny1 lead to usually large cells that are temperature-sensitive for growth in yeast (MacIntosh et al., 2001). Also, the sizes of small RNA for tRFs were much larger than the dicer-processed ∼22 nt ones, supporting the speculation of different endonuclease(s) in action. Therefore, generating Rny1 and RNase L mutants to assess any disruption in the fungal growth and development and most importantly quantify the changes in the levels of tRFs will answer the pending questions brought up by this study. Moreover, the biological function of this under-characterized class of small RNA in this major pathogen of all dicots demands further study. Questions such as whether tRFs are induced by virus infection or simply a stress response, whether they target the IGRs as Blastn results suggested, or whether tRFs can be manipulated to debilitate *S*. *sclerotiorum* remain to be answered in the future.

The results derived from this study pave the way for the development of new control strategies that exploit RNA silencing mechanisms. The external RNA pesticide developed suggests the occurrence of external uptake of RNA in *S*. *sclerotiorum*. Furthermore, host-induced gene silencing (HIGS), virus-induced gene silencing (VIGS) approaches or heterologous expression of dsRNA sprays (spray-induced gene silencing) targeting *agl*-2 in *S*. *sclerotiorum* are expected to be effective in reducing the virulence, adding to the tool box of disease control.

## Author Contributions

AN, PM, and S-YLM conceived and designed the experiments and wrote the manuscript. PM, CF, and HS performed the experiments. AN analyzed the small RNA data.

## Conflict of Interest Statement

The authors declare that the research was conducted in the absence of any commercial or financial relationships that could be construed as a potential conflict of interest.
