# Supplementary material for: Roles of Argonautes and Dicers on Sclerotinia sclerotiorum Antiviral RNA Silencing
Source: Front Plant Sci. 2019 Jul 30;10:976. doi: 10.3389/fpls.2019.00976 (PMC6694225; doi:10.3389/fpls.2019.00976)
Supplement: Supplementary file 3 [file Presentation_1.pptx]

## Slide 1
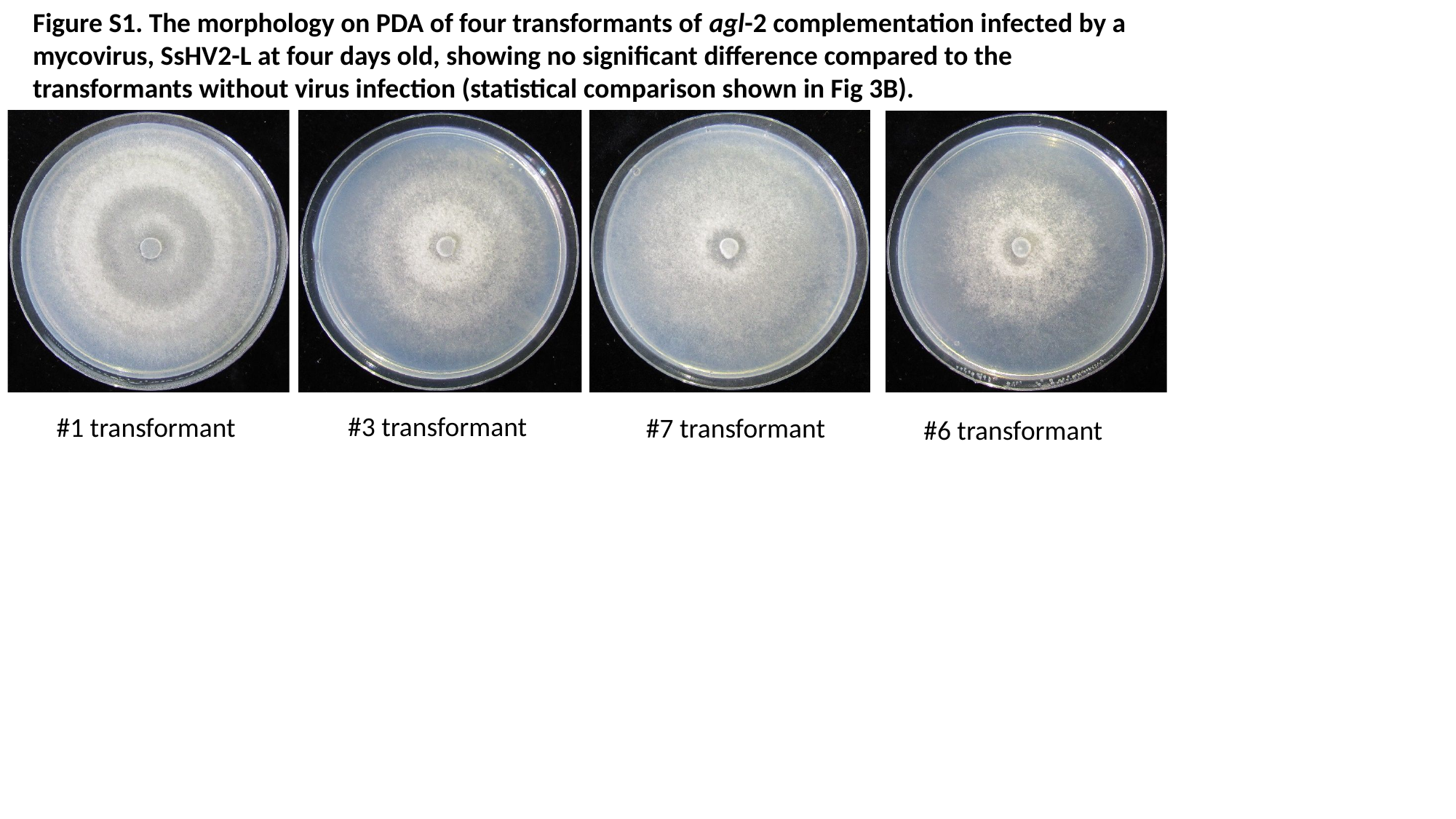

Figure S1. The morphology on PDA of four transformants of agl-2 complementation infected by a mycovirus, SsHV2-L at four days old, showing no significant difference compared to the transformants without virus infection (statistical comparison shown in Fig 3B).
#3 transformant
#1 transformant
#7 transformant
#6 transformant
