# Supplementary material for: Roles of Argonautes and Dicers on Sclerotinia sclerotiorum Antiviral RNA Silencing
Source: Front Plant Sci. 2019 Jul 30;10:976. doi: 10.3389/fpls.2019.00976 (PMC6694225; doi:10.3389/fpls.2019.00976)
Supplement: Supplementary file 4 [file Presentation_2.pptx]

## Slide 1
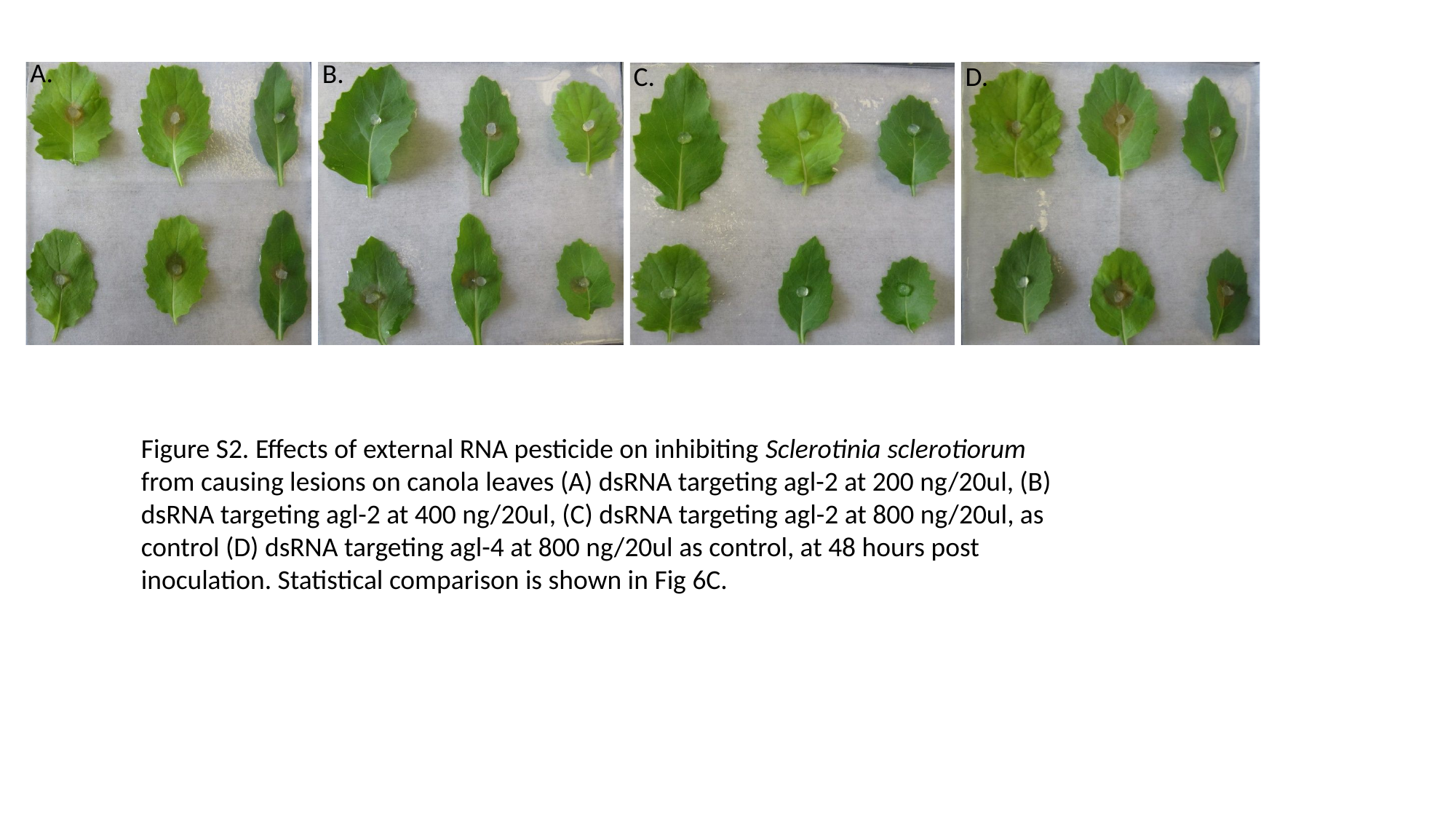

A.
B.
C.
D.
Figure S2. Effects of external RNA pesticide on inhibiting Sclerotinia sclerotiorum from causing lesions on canola leaves (A) dsRNA targeting agl-2 at 200 ng/20ul, (B) dsRNA targeting agl-2 at 400 ng/20ul, (C) dsRNA targeting agl-2 at 800 ng/20ul, as control (D) dsRNA targeting agl-4 at 800 ng/20ul as control, at 48 hours post inoculation. Statistical comparison is shown in Fig 6C.
